# Role of haptic feedback technologies and novel engineering developments for surgical training and robot-assisted surgery

**DOI:** 10.3389/frobt.2025.1567955

**Published:** 2025-06-04

**Authors:** Imán Laga Boul-Atarass, Mercedes Rubio Manzanares Dorado, Andrés Padillo-Eguía, Jesús Racero-Moreno, Ignacio Eguía-Salinas, Sheila Pereira-Arenas, Rosa María Jiménez-Rodríguez, Javier Padillo-Ruiz

**Affiliations:** ^1^ Department of General Surgery, Hospital University Virgen del Rocío, Seville, Spain; ^2^ Oncology Surgery, Cell Therapy and Organ Transplantation Group, Institute of Biomedicine of Seville (IBiS), Virgen del Rocio University Hospital, University of Seville, Seville, Spain; ^3^ Higher Technical School of Engineering, University of Seville, Seville, Spain

**Keywords:** biomedical engineering, 3D modeling, haptic feedback, haptic technologies, robotic surgery, surgical simulations, surgical training

## Abstract

Haptic feedback, or tactile perception, is presented by many authors as a technology that can greatly impact biomedical fields, such as minimally invasive surgeries. Laparoscopic interventions are considered the gold standard for many surgical interventions, providing recognized benefits, such as reduced recovery time and mortality rate. In addition to this, the advances in robotic engineering in the last few years have contributed to the increase in the number of robotic and tele-operated interventions, providing surgeons with fewer hand tremors and increased depth perception during surgery. However, currently, both techniques are totally or partially devoid of haptic feedback. This added to the fact that the skill acquisition process to be able to use these technologies shows a pronounced learning curve, has propelled biomedical engineers to aim to develop safe and realistic training programs using simulators to address surgical apprentices’ needs in safe environments for the patients. This review aims to present and summarize some of the latest engineering advances reported in the current literature related to the development of haptic feedback systems in surgical simulators and robotic surgical systems, as well as highlight the benefits that these technologies provide in medical settings for surgical training and preoperative rehearsal.

## 1 Introduction

Nowadays, laparoscopic surgery is a minimally invasive procedure considered the gold standard approach in many surgical interventions due to the many advantages that the technique provides, such as reduced recovery time and lower mortality rates (Kawka et al., 2023). However, laparoscopic training shows a pronounced learning curve that enhances the need for safe training programs for the patients that are also effective for the surgical apprentices outside the operating room (Kim et al., 2021; Braga et al., 2012). This surgical technique requires a deep understanding of laparoscopic instrumentation and a training period with diverse experiences and scenarios so that the trainee can be aware of the possible complications and learn how to prevent and treat them (Tang et al., 2017). The acquisition of these skills is challenged by the restricted working hours that limit the process of teaching and learning minimally invasive techniques (Sparn et al., 2024).

Following the example of the aviation industry, in which simulations are used to achieve the eminent technical skills required, along with a very small margin of error, technological advances in computer-aided simulations are also being applied to laparoscopic training (Vitish-Sharma et al., 2011). The creation of virtual environments provides the opportunity to recreate tailored and risky surgical situations without real-life repercussions, so surgeons can mitigate skill decay over time and trainees can improve according to their personalized needs and increase their confidence in their surgical skills (Nassar et al., 2021; Lohre et al., 2021; Mao et al., 2021). Considering that a hundred cases may be required for appropriate learning of the most complex procedures, virtual and computer-based simulations provided by biomedical engineers may be a good source of unlimited training cases (Susmitha et al., 2015).

In addition to this, laparoscopic surgery can benefit greatly from simulations since surgical instrumentation differs from the one used in conventional open surgery. Furthermore, psychomotor practice of complex maneuvers is required before the actual surgery to prove sufficient surgical competence to participate in human interventions, where margins of error are very small (Cardoso et al., 2023). Some of the required dexterities include aspects such as appropriate depth perception, hand-eye coordination, or bimanual manipulation (Vitish-Sharma et al., 2011; Sinha et al., 2017). For these reasons, the usage of these simulators would optimize the training time inside the operating room, where once the technical side is assimilated, the teachings can be more focused on decision-making training and intraoperative complications treatment (Matwala et al., 2024; Selva Raj et al., 2024).

Current laparoscopic simulators, such as the LapMentor™ (Simbionix USA Corp. Cleveland, OH) provide basic skill training, procedural tasks, simulation of full procedures, and feedback upon completion regarding parameters such as efficiency, accuracy rate, or safety parameters. Simulators usually provide performance curves that can be used to optimize training by tracking improvements and tackling specific weak points (Olivas-Alanis et al., 2020; Atesok et al., 2017).

Robotic surgery, on the other hand, provides high accuracy when performing repetitive tasks, with the additional advantage of telepresence, where a master console controls the slave robot that executes the command (Biswas et al., 2023). Surgical robots are composed of articulated instrumentation that accurately reproduces surgeons’ movements, overcoming the limitations imposed by the long and rigid instruments used in laparoscopic surgery. In addition to this, they provide relevant benefits, such as precise movements without tremors or improved visualization, which have contributed to their increased use over the years (Meli et al., 2017; Goldberg and Guthart, 2024; Mertz, 2022).

The ongoing engineering developments and applications of surgical robots are reflected in the percentage of robotic surgeries performed in the last years, which went from 1.8% in 2012 to 15% in 2020 (Sheetz et al., 2020). The most prevalent robotic surgical system is the da Vinci robot (Intuitive Surgical Inc.), created in 2000 with four different generations developed over the last 20 years, and the first to have FDA approval for surgical applications (Hamdi et al., 2024).

Other robot-assisted surgical systems are: the Arthrobot, developed in 1983 and considered the first robot to assist a surgical procedure in history (The Medical Post, 1985); the Puma robot, developed in 1985, used to perform a brain biopsy (Kwoh et al., 1988) and a transurethral prostate resection (Davies et al., 1991); and the ROBODOC, developed in 1991, and involved in assisting in implant positioning (Taylor et al., 1999).

However, although in laparoscopic surgery, the tactile perception, or “haptic feedback,” is severely limited by the interaction between laparoscopic instruments and the patient, in robotic surgery, the telemanipulation and the physical isolation of the surgeon from the patient worsen even more this sensory loss since direct contact between these is nonexistent (Meccariello et al., 2016). Therefore, the lack of haptic feedback is currently the main limitation of robotic surgical systems, especially since it is considered a key element to increase performance in a wide variety of tasks, such as robotic catheter insertion, palpation, or microneedle positioning (Meli et al., 2017; Najafi et al., 2023). In addition to this, the lack of haptic feedback also leads to excessive force application when using robotic systems for inexperienced surgeons (Jourdes et al., 2022).

Haptic feedback can be divided into kinesthetic and cutaneous. While the first one is related to the forces applied to joints and muscles, the latter is focused on tactile sensations associated with the skin. Tactile haptics refers to the stimulation of tactile sensing through haptic devices, which evokes the real feeling when touching an object (Selim et al., 2024; Abiri et al., 2019a).

Under the term “haptics”, several magnitudes are included, such as pressure, forces, texture, temperature, or vibrations (Shi and Shen, 2024). In humans, the sense of touch requires a combined activation of both tactile and kinesthetic force feedback through mechanoreceptors in skin and muscles, respectively. However, contrary to what happens in open surgery, in minimally invasive interventions, sensory perception is limited to the interaction between the tissues and the instrumentation used (Jourdes et al., 2022). While in open surgery surgeons rely on their fingertips’ sensations, in minimally invasive surgery all the sensory feedback comes from the tip of the tool that the surgeon uses.

Most minimally invasive devices lack haptic feedback, and physicians deal with an absent touch perception that difficult crucial tasks, such as tissue manipulation (Selim et al., 2024; Abiri et al., 2019a) since surgeons make use of their sense of touch to locate hidden structures and to distinguish abnormal tissues based on their altered mechanical properties in comparison to the healthy adjacent ones. Many researchers have focused on developing tactile feedback systems using vibrational or pneumatic stimuli to activate skin mechanoreceptors to improve surgical performance (Abiri et al., 2019a). Therefore, it is critical to dispose of devices capable of delivering haptic feedback during training, in the case of simulators, and intraoperatively, in the case of robotic surgeries (Abiri et al., 2019b).

Furthermore, considering that tactile sensations are essential in surgical fields, the archetype of a surgical simulator should provide an immersive experience, with the same stimuli and sensations that are encountered in the operating room (Di Vece et al., 2021). Current simulators come with some degree of feedback for the trainee. This feedback can be augmented feedback, referring to the information or total score that the users receive according to their technique (patterns, incorrect movements…), or intrinsic feedback, related to the sensorial stimulation that the trainees experience while using the simulator (haptics, audiovisual…) (Nassar et al., 2021). In addition to this, the main components responsible for haptic feedback are sensors and actuators. While sensors act by detecting the forces that the user applies to the tissues through the instrumentation, actuators transmit this information to surgeons’ hands (Abiri et al., 2019b).

Surgeons usually compensate for this lack of haptic feedback by increasing their training level and experience, and by focusing on intraoperative visual cues. However, this may lead to longer interventions and increased risk of complications than if haptic feedback were perceived similarly to in open surgery (Pinzon et al., 2016). Considering that the perception of an object’s mechanical properties requires a combination of visual and haptic information, motor task performance can be greatly improved by integrating haptic feedback that complements the already existing visual information (Pinzon et al., 2016).

In light of the above, this review aims to provide insights into the benefits and limitations of laparoscopic simulators and robotic surgery, the recent bioengineering developments in haptic feedback integrations, and their potential impact on training and procedure outcomes.

## 2 Benefits of laparoscopic simulators in medical training

Several authors have explored the utility of simulators for medical training. For instance, the impact of computer simulators on surgical skills was evaluated on a 3-week training program on a LapMentor™ simulator that included residents and medical students with basic to no laparoscopic experience (Kim et al., 2009). The authors showed that residents with some laparoscopic background initially benefited the most from the program, with faster acquisition and accuracy of the learned techniques, evaluated according to each task’s total transit time and accuracy. However, in the last stages of the interventional program, medical students improved significantly to almost reach the residents’ level of proficiency (Kim et al., 2009). These findings seem to indicate that, regardless of initial laparoscopic experience, computer-based simulators can help in a substantial acquisition of surgical techniques within a short time frame.

In addition to this, another study carried out a 4-week training intervention on 21 surgical residents using a LapMentor™ simulator (Andreatta et al., 2006). Once the program was completed, they evaluated participants’ improvement by putting into practice some laparoscopic skills in male pigs. The author reported a more accurate and precise 30° camera navigation in comparison to the control group as well as better ambidexterity abilities. The clinical consequences of this improved performance were less peripheral organ injury and decreased rates of untargeted electrocautery damage.

Important aspects of these simulators are their predictive validity, which refers to how reliably the real-life proficiency can be predicted according to the surgeon’s score and performance on the simulator, and their construct validity, which appropriately distinguishes experienced from inexperienced surgeons according to their score on the simulator. These parameters were evaluated by some authors, who correlated motion analysis data obtained from the LapMentor™ with the outcome of video assessments and the surgeon’s experience. They reported that the LapMentor™ distinguishes novices from experienced laparoscopic surgeons, and also that those with accurate performances on the simulator also executed safe laparoscopic procedures (Matsuda et al., 2012).

Some authors compared the efficiency of the LapMentor™ in comparison to a box trainer [Large Body MITS (TRLCD05)]. After 3 h of training for each group, the authors described an increased safe performance in both groups, which was higher in the group trained with the LapMentor™ simulator. The authors evaluated path length, tissue handling, and how the trainees were able to maintain the surgical instruments within the field of vision, and suggested that a combination of both methods may lead to a reduction in the learning curve and better laparoscopic training (Vitish-Sharma et al., 2011). In addition to this, simulators provide additional benefits lacking in box trainers, such as personalized feedback or complex procedure simulations (Våpenstad et al., 2013).

A more broad-ranging study was performed of three different simulators: LapSim® (Surgical Science Sweden AB, Gothen ® burg, Sweden), LAP Mentor III® (Simbionix, Tel Aviv, ® Israel), and LaparoS® (VirtaMed AG, Zurich, Switzer land). The authors showed faster task completion and a reduced path length in tasks such as bimanual handling, clip application, and tissue dissection. Moreover, they also reported that remarkable improvements can be achieved in virtual laparoscopic training regardless of the type of simulator employed (Sparn et al., 2024).

## 3 Value of haptic feedback technology in simulators for surgical training

In addition to appropriate training, touch sensations are also extremely important for surgical performance. It has been reported in the literature that better performance and higher learning rates are observed in trainees who were exposed to haptic feedback during their training stages than those who were not trained using haptic feedback. This improvement is especially remarkable in the early stages of learning (Zhou et al., 2012).

Since the haptic feedback in current simulators is not yet well developed, most of the laparoscopic learning process is spent on adapting to the loss of physical cues and their replacement with visual ones (Trute et al., 2024). Surgeons are already so used to this sensory substitution that some studies reported that when experienced surgeons undergo laparoscopic training without haptic feedback, their performance does not seem to be greatly affected. This is a consequence of their vast experience, which allows them to replace the haptic feedback with visual cues learned during their careers (Pinzon et al., 2016).

Nevertheless, many bioengineering studies are working on mimicking tactile feelings using haptic devices and on simulating as realistically as possible both the appearance and the interactions between the different instruments and the tissues involved (Table 1).

**TABLE 1:** Relevant studies included in this review about haptic feedback in simulators.

Reference	Year	Region	Participants	Engineering device	Instruments	Haptic type	Tasks	Measurements
Zhang et al. (2023)	2023	Canada	10 novices	Phantom Omni (Senseable Technologies)	Grasper Laparoscopic scissors	Unspecified	Pattern-cutting tasks inside a training box	• Total task time • Total grasping time • Total scissor time • Total scissor cutting time • Number of haptic feedback instances • Cutting accuracy
Våpenstad et al. (2013)	2013	Norway	20 surgeons	LapSim (Surgical Science AB)	Xitact IHP Xitact ITP	Force	Fine dissection Lifting and grasping	Questionnaires related to the perception of the handles
Zhou et al. (2012)	2012	United States	20 novices	ProMIS MIST-VR	Laparoscopic tools	Unspecified	Suturing and knot-tying	• Time to task completion • Instrument path • Instrument smoothness • Errors
Di Vece et al. (2021)	2021	Italy United States	14 urologists and students	CHAI3D OpenHaptics™	Veress Needle controlled by a haptic device stylus	Tactile	Access the abdominal cavity twice	• Insertion error • Duration of the task • Number of mistakes
Alleblas et al. (2019)	2019	Netherlands	11 surgeons	Force Reflecting Operation Instrument (FROI)	Laparoscopic grasper	Unspecified	10 partial bowel resections 6 hemihysterectomies 2 ovariectomies 6 partial ureter dissections	Questionnaire (NASA Task Load Index)
Alleblas et al. (2016)	2016	Netherlands	279 gynecologists, general surgeons, urologists, pediatric surgeons and medical technicians	Laparoscopic tools	Scissors In-line handles Pistols	Unspecified	Obtain expert opinions regarding handle designs and expectations towards haptic feedback instruments	Questionnaires assessing: • Handgrip assessment • Haptic feedback
Botden et al. (2008)	2008	United Arab Emirates Netherlands	45 surgical residents	SimSurgery Simulator Box trainer	Xitact HTP instrument ports	None	Two-handed stitch with traction Realistic surgeon’s knot Realistic interrupted suture Realistic free knot	Parameters of the final laparoscopic knot: • Position of the needle in the holder • Running needle through suturing pad • Taking proper bites of the suturing pad, during suturing • Throwing thread around a holder • Pulling tight of the thread • Quality (strength) of knot • Global evaluation of performance
Quesada et al. (2018)	2018	Spain France	—	OpenHaptics Toolkit	Laparoscopic grasper	Visual Force	Tearing of soft tissues	Computing times
Kim et al. (2013)	2013	South Korea	—	Custom-designed device	Conventional laparoscopic instruments	Force	Gallbladder removal	Computation time
Wu et al. (2022)	2022	China	—	Geomagic Touch	Coagulation hook Grasping forces Titanium clamps	Force	Clipping procedure Disjunction of vessels	The time cost of each step
Hamza-Lup et al. (2012)	2012	Romania United States	—	Phantom Omni (Senseable Technologies)	Tools for palpation (e.g., Babcock grasper, Maryland grasping forceps)	Force	Liver palpation	• Questionnaires • Forces applied to the liver
WU et al. (2021)	2021	China	—	Geomagic Touch	Forceps Scissors	Visual Force	Hepatic parenchymal removal	• Time cost of the simulation cycle • Geometrical information
Boutin et al. (2024)	2024	Canada	30 surgical residents	SemseGlove NOVA haptic gloves	Surgical drill	Vibrotactile Force	External ventricular drain placement	• Questionnaire • Speed and accuracy of the procedure
Heredia-Pérez et al. (2019)	2019	Mexico Japan	5 expert neurosurgeons 11 surgical residents	PHANTOM PREMIUM 1.0, Geomagic	Forceps	Force	Removal of transsphenoidal tumor	• Colission point report • % healthy and tumor tissue removed • Total time of task completion • Path of virtual tools • Frequency of grasping action • Frequency of foot pedal activation
Lin et al. (2014)	2014	China	9 experienced surgeons 16 novices	Omega.6 (Force Dimension)	Saw Other tools for craniomaxillofacial surgery	Force	Bone-sawing procedure	• Operative time • Haptic forces of the process • Feed velocity • Acceleration of the tool
Heimann et al. (2021)	2021	Germany United States	—	TouchX (3D Systems)	Forceps Syringe Straight instruments (scalpel, cotton tip) Special tool for implant release	Visual	Insertion and refill of an eye implant for intravitreal drug delivery	—

To achieve this, some authors focused their efforts on gathering information about this tissue-tool interaction and how it is perceived by surgeons to develop an accurate model. They evaluated surgeons’ perception of tissue stiffness when using laparoscopic instruments and compared their subjective opinions with laboratory measurements (Pinzon et al., 2016). The authors reported that the bigger the amount of tissue held in the grasper, the more accurate their stiffness assessment. Moreover, they also proposed four parameters responsible for the differentiation among several tissue types, which are the mass of the tissue, the mass of the tissue held by the laparoscopic instruments, tissue stiffness, and the degree of attachment to the abdominal wall (Pinzon et al., 2016).

Regarding the value of haptic feedback, previous work proposed suturing as a surgical task that benefits greatly from the tactile sensation of the tissue tension and suture thread tightness. Since the interactions between the different elements (needle, tissue, thread, instruments…) were so important, trainees did not benefit from the available simulations at that time without any haptic feedback, and participants reported a preferred use of box trainers instead (Botden et al., 2008).

**FIGURE 1:**
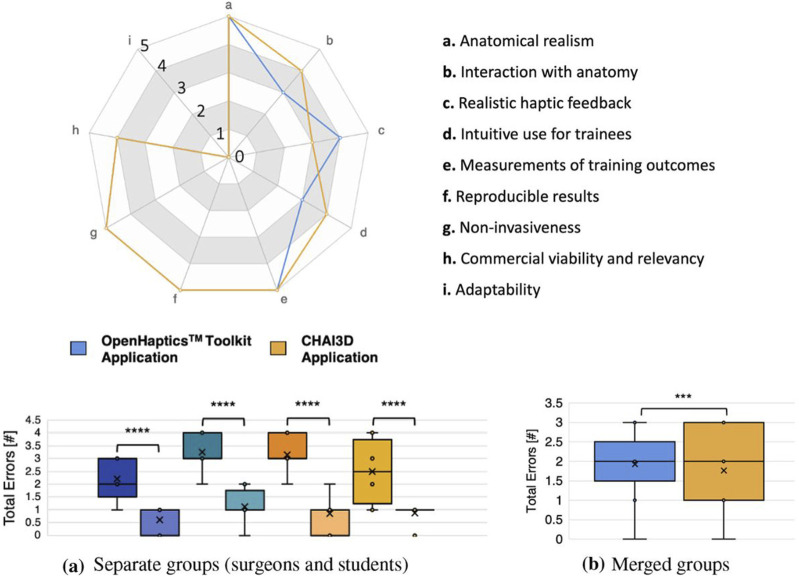
Qualitative features of two simulation platforms, the OpenHapticsTM and the CHAI3D (upper panel). Boxplots showing performance of surgeons and students at two different attempts, using the simulation platforms (lower panel). Image modified from Di Vece et al. (2021). **(a)** Seperate groups (surgeons and students). **(b)** Merged groups.

However, a posterior study showed that haptic feedback is more relevant in some force-related tasks than in orientation-based procedures that require precise gestures and instrument control, such as suturing or knot-tying (Zhou et al., 2012). This reinforces the results of a recent publication, in which haptic feedback is presented as especially relevant for some crucial laparoscopic tasks, such as the abdominal insertion of the needle (Di Vece et al., 2021). This procedure heavily relies on tactile perception as the needle is inserted across the layers of the abdominal wall. The authors developed a simulator called OpenHapticsTM to evaluate the combined benefit of the combination of virtual simulations and haptic feedback for an appropriate insertion of the Veress needle without internal organ damage (Figure 1) (Di Vece et al., 2021). In this line of thought, some authors also explored the influence of force feedback on the amount of exerted force during dissection tasks using a master-slave device. They found a decrease in the applied forces when haptic feedback was present (Pinzon et al., 2016).

**FIGURE 2:**
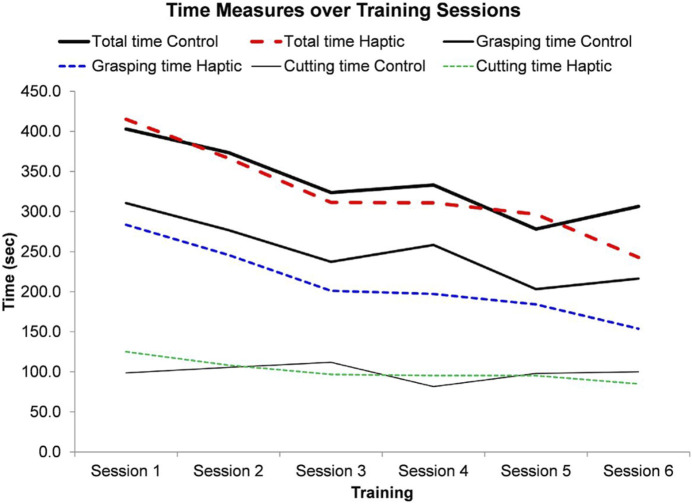
Time measurements over six training sessions. Image obtained from Zhang et al. (2023).

Considering the importance of haptic feedback, to further enhance training using devices with this property, a very recent work aimed to develop a dual-user simulator and achieve haptic feedback transfer from one user (e.g., an experienced surgeon) to another (e.g., a trainee), and guide novices’ hand movement based on the maneuvers executed by the expert surgeon. The authors of this work reported improved learning when haptic feedback between users was involved, and when trainees were assisted in simple executive tasks with laparoscopic tools, although further studies are required for more complex procedures (Figure 2) (Zhang et al., 2023).

In addition to this, recent improvements are being made in the development of new haptic laparoscopic tools. For instance, a haptic laparoscopic grasper called the Force Reflecting Operation Instrument (FROI) was created using optical fiber Bragg grating technology and provides some resistance at the level of the tool trigger, as well as an audio warning and a mechanical brake that is activated when excessive forces are exerted over the tissue (Alleblas et al., 2019).

In this line of thought, some authors have previously explored the ergonomic aspects of laparoscopic handle design. They reported a considerable number of physical complaints from experienced surgeons associated with the use of laparoscopic instruments (i.e., the standardized size of the tools), which can lead to excessive force application and unfavorable postures (Alleblas et al., 2016). This highlights the importance of ergonomically designed handles that may optimize the implementation of laparoscopic tools with haptic feedback.

The value and significance of simulators have been enhanced in the last years, after the COVID-19 pandemic. A few years ago, the Royal College of Surgeons of England (RCSE) published a report after the pandemic about how technology may help to identify trainees’ needs and enhance surgical training, especially in those situations (i.e., a pandemic) where the number of surgical interventions is reduced to the bare essentials and no real practice is possible. The RCSE reported that virtual solutions may help assess surgical competence and involvement in surgical training. Furthermore, they stated that although haptic feedback may increase the applicability of simulation technologies, further research is still required for its optimization (Adebayo et al., 2022).

In light of the above, it is evident that laparoscopic simulators are a potent tool to refine surgical competence outside of the operating room and achieve a proper transfer of the learned skills to real-life clinical scenarios. However, the application of this technology in clinical frameworks may be cost-prohibitive, and open centralized training centers may help to overcome this limitation. In addition to this, the implementation of haptic feedback in simulation technologies is challenged by the need for realistic modeling of the elements involved in a surgical intervention (i.e., tools, organs) and the interactions between them, as well as the computational costs that these calculations require (Jourdes et al., 2022). Moreover, tissue simulations are often based on laboratory measurements from cadaveric samples, which makes the biomechanical properties simulated (Young’s modulus and Poisson’s ratio) unrealistic, and require more *in vivo* measurement approaches.

## 4 Robotic surgery and haptic feedback integration

Robotic surgery arose as a technology aimed at overcoming some of the limitations of laparoscopic surgery, such as hand tremors, poor depth perception, or the impossibility of telepresence. However, while laparoscopic techniques provide surgeons with some extent of haptic feedback, robotic surgery suffers from a complete loss of it. In addition to this, robotic surgical systems are more complex and entail some costs related to their acquisition and maintenance (Bergholz et al., 2023), as well as others associated with disposable supplies, such as trocars or drapes (Feldstein et al., 2019). This makes robotic surgery considerably more expensive than conventional minimally invasive surgery (Hamdi et al., 2024; El Rassi and El Rassi, 2020). The technical complexity, the economic costs, and the need to modify current instrumentation are the main limitations of haptic feedback integration with current robotic technologies in surgical settings (Hamdi et al., 2024). Table 2 shows the most relevant works on robotic surgery and haptic feedback included in this section.

**TABLE 2:** Relevant studies included in this review about haptic feedback in robotic surgery.

Reference	Year	Region	Participants	Engineering device	Instruments	Haptic type	Tasks	Measurements
Hamdi et al. (2024)	2024	Saudi Arabia Japan	—	A custom robotic master-slave	Custom robotic finger	Vibrotactile	Interact with 3 surfaces of different materials	Joint motion from: • metacarpophalangeal master-slave • proximal interphalangeal master-slave • wrist master-slave
Golahmadi et al. (2021)	2014	Australia	20 subjects	A robotic master-slave system (PRAMiSS)	Camera Phantom Desktop (Sensable Technologies) Laparoscopic instrument	Visual Force	Vision feedback for tissue stiffness characterization Force feedback for tissue stiffness characterization Vision and force feedback for tissue characterization Direct exploration of tissue	Questionnaires (True/False answers)
Ouyang et al. (2021)	2021	Germany	31 inexperienced subjects	A robotic master-slave system (FLEXMIN)	Intracorporeal single-port instrument	Force	Precise moving and positioning of the instrument tip	• Applied touching forces • Total times
Ueda et al. (2023)	2021	China United States	15 novices	Vinci Robotic Surgical System (Intuitive Surgical, Sunnyvale, CA)	Surgical forceps	Visual Bio-inspired	Phantom palpation to locate a tumor	• Accuracy of the localization • Mean force • Time to completion • Tumor contact time
Abiri et al. (2019b)	2019	United States	15 novices	Vinci IS 1200 surgical system	da Vinci Fenestrated Bipolar forceps	Force Pneumatic Kinesthetic Hybrid Kinesthetic-Tactile	Single handed peg-transfer tasks	• Average grip force • Peak grip force • Number of faults
Di Vece et al. (2021)	2019	United States	19 subjects (task 1) 14 subjects (task 2)	Da Vinci robot (Intuitive Surgical, Sunnyvale, CA)	da Vinci Fenestrated Bipolar forceps	Force Vibrotactile	Detection of tubular structure Detection of discrete tumor	• Correct localization • False localization • Time of completion
Meccariello et al. (2016)	2016	Italy Saudi Arabia	25 surgeons (Otolaryngology, Thoracic Surgery, General Surgery, Urology and Gynecology)	Vinci Si Robotic Surgical System (Intuitive Surgical, Sunnyvale, CA)	Maryland forceps A monopolar rounded tip	Visual	Identify consistency or softness of tissue Locate a small metallic clip	Questionnaire
Jourdes et al. (2022)	2022	France	1 robotic surgeon	Vinci Xi surgical system (Intuitive Surgical) Simulator	Needle Scissors Grasper	Visual	Knot-tying and suturing tasks	• Thread behavior tests • Stress of tissue • Stress on rigid objects

The lack of haptic feedback in robotic surgery, along with the inherent capacity of surgical robots to exert strong compressive and shear forces, has led to an increased risk of surgical mistakes during blunt dissection tasks and intraoperative tissue injuries. In the absence of haptic feedback, previous studies have reported that forces of only 1.25 N can cause tissue damage (Abiri et al., 2019a). In this regard, some authors have presented design frameworks aimed at providing an adjustable and constant force to ensure safe tissue maneuvering during minimally invasive robotic interventions in the absence of haptic feedback (Cheng et al., 2023; Sun and Lueth, 2023).

In addition to this, the importance of haptic feedback in tissue handling was also evaluated in a study in which haptic feedback was incorporated in a Parallel Robot Assisted MIS System (PRAMISS) that measured tissue interaction forces at the tooltip and achieved a proper attenuation if these were excessive (Moradi Dalvand et al., 2014). Moreover, authors of a recent evaluation reported 22.7% more force application by novices than experienced surgeons. They also showed that trainees benefit from feedback mechanisms, leading to a 47.9% decrease in the exerted forces (Golahmadi et al., 2021).

**FIGURE 3:**
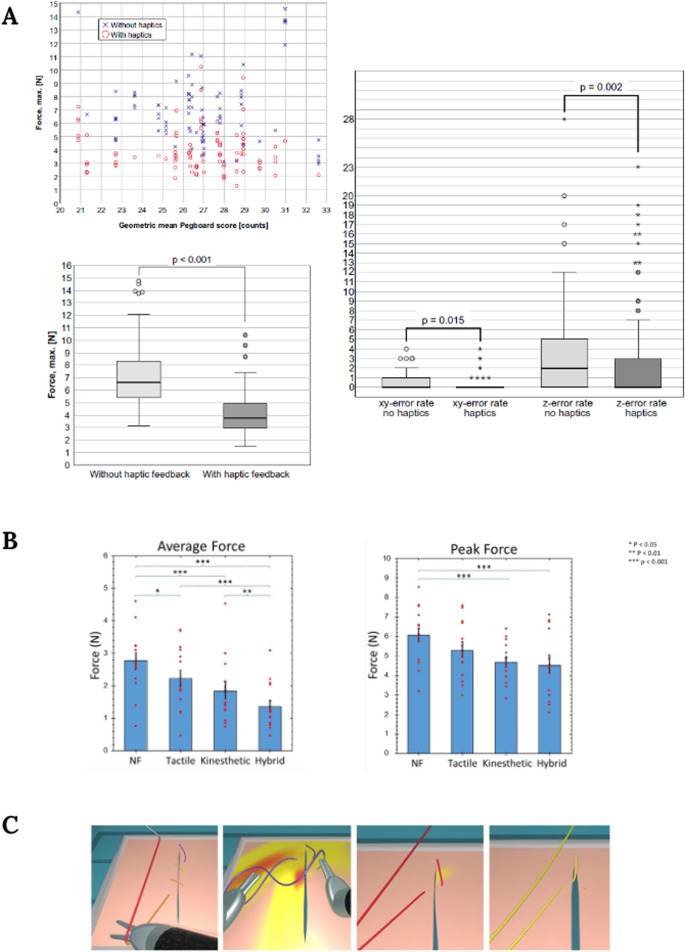
**(A)** Graphs showing force exertion (left), and error rates (right) with and without haptic feedback. Image obtained from Miller et al. (2021). **(B)** Performance in terms of average and peak grip force under different feedback situations: no feedback (NF), tactile, kinesthetic, and hybrid feedback. Image obtained from Abiri et al. (2019a). **(C)** Stress gradients representation on thread and tissue when excessive force is applied. Image obtained from Jourdes et al. (2022).

In a similar study, authors developed a robotic master-slave system called FLEXMIN to evaluate the effect that tactile perception has on the forces applied using the surgical robot. In this work, the authors also reported a significant reduction in the exerted forces when haptic feedback was present (Figure 3A) (Miller et al., 2021).

Considering that the actuators transmit information to surgeons’ hands according to what the sensor in the robotic instruments detects, the conversion process of the information from the sensor to the actuator is the main factor to address to provide natural haptic feedback that consists of more than just vibration or pressure. Some authors aimed to develop bioinspired algorithms that convert the information received by the sensors in a similar way that rapidly adapting type 1 neurons and Pacinian corpuscles do in the skin to increase performance. With this approach, they achieved less force exertion and better localization of soft tumors (Ouyang et al., 2021).

**FIGURE 4:**
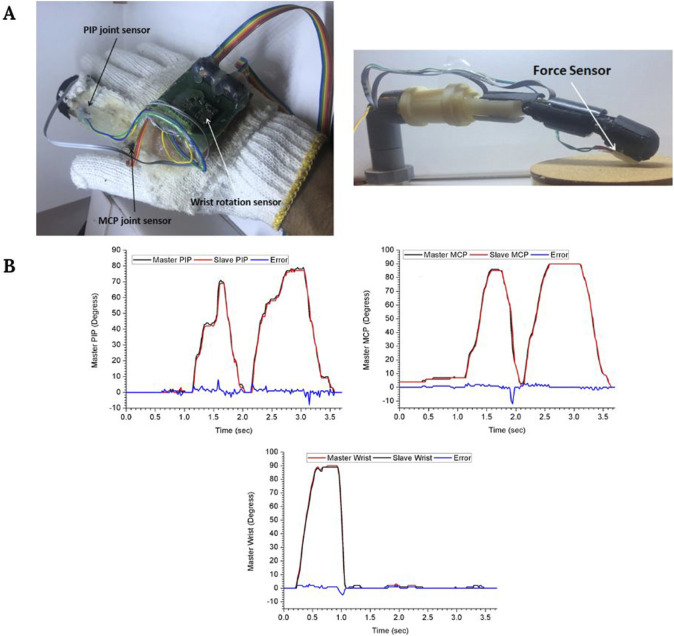
**(A)** Master glove and robotic finger. **(B)** Master and slave motion at the proximal interphalangeal (PIP) joint, the metacarpophalangeal (MCP) joint, and the wrist. Figure modified from Hamdi et al. (2024).

In addition to this, other recent works have been reported progressing towards the development of haptic sensations in robotic surgery. For instance, some authors are developing a wearable glove (Figure 4A) with robotic surgical fingers that use vibrational amplitude differences to help surgeons distinguish between hard, firm, and soft surfaces (Hamdi et al., 2024). Additionally, they also report a good correlation between the surgeon’s and the robotic finger’s movements (Figure 4B). On the other hand, other studies propose alternative mechanical feedback approaches, such as pneumatic balloons for the da Vinci robot that stimulate surgeons’ mechanoreceptors (Abiri et al., 2019b). This is an example of how some authors are also exploring the incorporation of analog or hybrid haptic solutions to avoid adding complexity to the already complex robotic devices. Additionally, these hybrid implementations (Abiri et al., 2019b; Ueda et al., 2023) are potentially less expensive than fully digital haptic systems, which is one of the main limitations of the acquisition of surgical robots.

However, despite the extensive research focused on the development of haptic feedback in robotic surgery in the last few years, current technologies still fail to replicate the natural touch feelings. This is a consequence of the current unimodal technologies, which focus on a single modality of feedback (tactile or kinesthetic) (Amirabdollahian et al., 2018). This approach is not well interpreted by the human brain, which is accustomed to receiving and integrating sensory signals from both pathways.

Therefore, multimodal haptic feedback systems that target skin and muscle mechanoreceptors constitute a promising approach to the current haptic feedback challenge in minimally invasive surgeries, both robotic and laparoscopic. Nevertheless, this line of action has not been widely explored, due to the technical limitations associated with the integration of several sensing and feedback modalities. Some authors explored this approach by developing a multimodal haptic feedback system that combined tactile and kinesthetic force feedback. In this study, the authors evaluated this multimodal system through the performance of two-handed peg transfer tasks and recorded the users’ grip force. With this multimodal system, authors reported greater benefits, especially in novice surgeons, in comparison with single modality systems. Among the observed advantages, authors described 50% less grip force than when haptic feedback is absent (Figure 3B) (Abiri et al., 2019a).

Additionally, the introduction of haptic feedback systems in robotic surgery is limited due to the requirements for modification in robotic instrumentation. Some authors have proposed employing multimodal haptic feedback sensors as add-ons to robotic tools, therefore allowing some degree of versatility and compatibility with several robotic systems available on the market. They evaluated the ability of this approach to accurately discriminate soft tissues and discern underlying structures through force and vibrotactile feedback and found that multimodal haptic feedback significantly increased the effectiveness of artificial palpation devices (Abiri et al., 2019b).

Since haptic feedback in robotic surgery is still far from developed, many studies have focused on understanding how robotic surgeons rely on visual cues to evaluate and interpret the surgical field, and how their expertise also may impact the interpretation of this visual information and therefore, the surgical outcome, when haptic feedback is nonexistent (Green et al., 2022; Hagen et al., 2008). In addition to this, some authors have also performed some comparisons between visual and haptic feedback in virtual reality environments, where users were found to perceive better the interactions when both modalities coexist, although haptic feedback alone was more effective than visual feedback alone (Gibbs et al., 2022). On the other hand, a different study performing a similar comparison using a robotic arm found that visual feedback modalities reduced the most the grasping force during object-grasping tasks (Haruna et al., 2021). Therefore, appropriate and comprehensive communication of visual information is key to robotic surgery training so that future surgeons can interpret visual signals of force application when working with insensitive surgical robots (Miller et al., 2021).

In addition to this, when haptic feedback is not possible, sensory substitution arises as a compensatory approach to provide surgeons with a different sensory modality to represent, for instance, the force applied by the surgical instruments (Meccariello et al., 2016), through graphical or audio feedback (Okamura, 2009). An example of this is a work in which, to replace haptic feedback in simulators, some authors developed a training approach in which as the users perform tasks related to suturing and knot-tying, they receive color-based feedback according to the stress fields computation performed in real-time (Figure 3C). This approach may help trainees acclimate to the lack of tactile feedback and prevent excessive force application (Jourdes et al., 2022). However, for other authors, sensory substitution is counterintuitive and unnatural, may worsen the learning curve, and does not provide any information related to hidden structures in the subsurface (Miller et al., 2021).

To sum up, future studies are still needed to tackle some important limitations of haptic feedback implementation in robotic surgery. One of them is the standardization and magnification of the force feedback perceived through the device (Pinzon et al., 2016). In addition to this, there is a pressing need for feedback systems that encode information in a way that emulates the human nervous system. A stable implementation should also be achieved without time delays and communication latency during processing and transmission tasks to minimize the risk of instability of the haptic loop and compromise the surgery, especially for intercontinental applications.

## 5 Case studies of novel haptic feedback developments for simulations and surgical robots

### 5.1 Abdominal surgery

Nowadays, the core problem that prevents current simulation technologies from adopting haptic feedback is the feedback rates, which need to be extremely fast (from 500 to 1 kHz) to achieve a realistic feeling. This implies a huge computational cost since nonlinear mechanics equations must be solved around 1,000 times per second. In surgical simulations, the cutting steps that require big changes in the mesh topology are especially hard to represent. For this reason, some authors have focused on the simulation of interventions for the cutting steps that are minimal, such as laparoscopic cholecystectomies, where only one step is properly considered as cutting, and where tissue tearing is a more frequent procedure. In a study, authors reported the development of a novel algorithm for simulating the tearing of the fat tissue that usually takes most of the intervention time, with reduced computation times, and is compatible with haptic implementations (Quesada et al., 2018).

**FIGURE 5:**
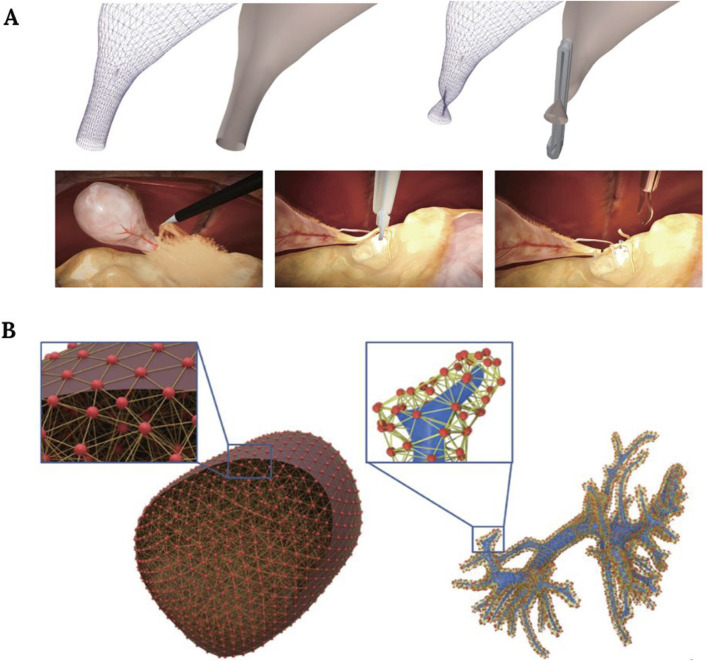
**(A)** Simulation of the clipping procedure during a cholecystectomy. Image modified from Wu et al. (2022). **(B)** Liver model illustration, showing the mesh and physical particles of the liver surface (left), and intrahepatic vessels (right). Image obtained from Wu et al. (2021).

There are three basic procedures in laparoscopic cholecystectomy: 1) Calot’s triangle dissection, 2) cystic artery clipping, and 3) gallbladder separation. Some authors used finite element methods to simulate the step related to gallbladder removal that consists of the separation from the liver by burning the surrounding connective tissue (Figure 5A) (Kim et al., 2013). On the other hand, another study went further and tried to simulate all three of them. Their approach facilitates both soft deformation and haptic rendering and uses a position-based dynamics method that overcomes the real-time limitation posed by the high computational cost of finite element methods, but with shortcomings related to the graphics and unrealistic visual results (Wu et al., 2022).

In liver surgery, preoperative palpation is an important task that can reveal multiple pathological conditions. With this in mind, some authors presented a simulation system in which these palpation skills can be learned, implementing a force feedback hardware interface and a deformable liver model (Hamza-Lup et al., 2012). A similar attempt was reported by authors who tried to simulate liver parenchymal transection (Figure 5B) (Wu et al., 2021).

In robotic interventions, the Senhance Surgical System (SSS) is an alternative to the da Vinci robot. It has been tested for robotic cholecystectomies, and some authors report good haptic force feedback perception. The SSS also offers additional benefits, such as eye-tracking camera control and high configuration versatility (Aggarwal et al., 2020; Melling et al., 2019).

In addition to this, some authors are working on novel robotic systems. For instance, a recently published study presented a surgical-assisting robot (Riverfield Inc., Japan) with bimanual haptic devices placed in the surgeon’s console. The device was tested in animal models to evaluate its impact on force reduction in cases with a high risk of intestinal damage (Ota et al., 2024).

### 5.2 Brain surgery

In brain surgery, the placement of a ventricular drain to relieve intracranial pressure is a common procedure that must be mastered by neurosurgical trainees. To help with this task, some authors developed a simulator combined with haptic gloves, the SenseGlove NOVA gloves, which incorporated vibrotactile feedback. The feedback intensity increased as the skull was penetrated by the surgical drill, and then dropped to indicate that the drill must be stopped immediately (Boutin et al., 2024).

Moreover, other complex surgical brain interventions also benefit from virtual simulators, such as the resection of pituitary tumors through a transsphenoidal procedure. A study reported the development of an intuitive simulator with haptic feedback that helped achieve finer movements with less undesired contact with healthy tissue (Heredia-Pérez et al., 2019).

**FIGURE 6:**
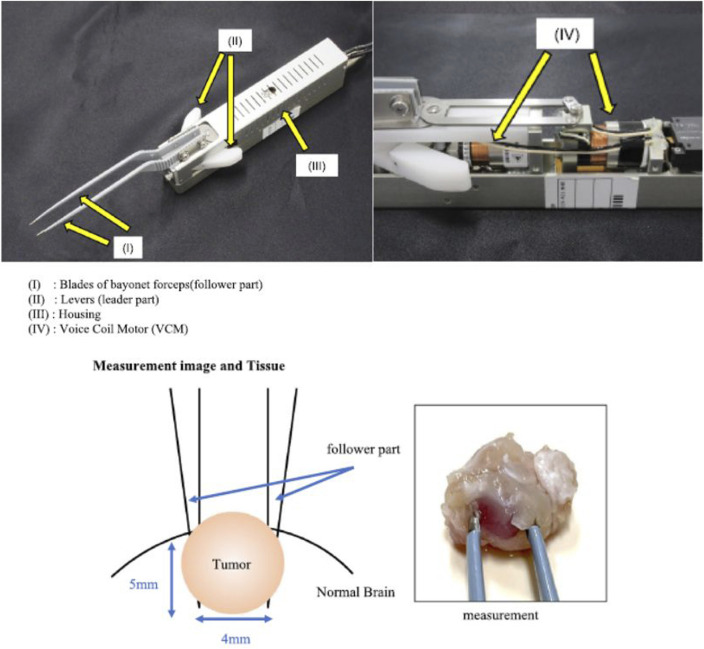
Surgical forceps with haptic technology for brain tissue stiffness measurement. Image modified from Ezaki et al. (2024).

In addition to this, during real-life brain surgery, it is of utmost importance to distinguish between tumor tissue and normal brain tissue according to stiffness differences. For this purpose, some authors developed a haptic forceps prototype and aimed to determine their clinical utility on mouse models of brain tumors (Figure 6) (Ezaki et al., 2024).

### 5.3 Bone surgery

In bone surgery, bone sawing in maxillofacial surgery is a complex procedure where surgeons must be sensitive to the force they apply in the operating room. Due to the relevance of this procedure, some authors aimed to develop a haptic simulation platform where bone-sawing skills can be assessed and validated. They employed multi-point collision detection methods to simulate tool-bone interactions, and considered bone density (cortical and trabecular), feed velocity, and spindle speed to simulate the haptic feedback (Lin et al., 2014).

**FIGURE 7:**
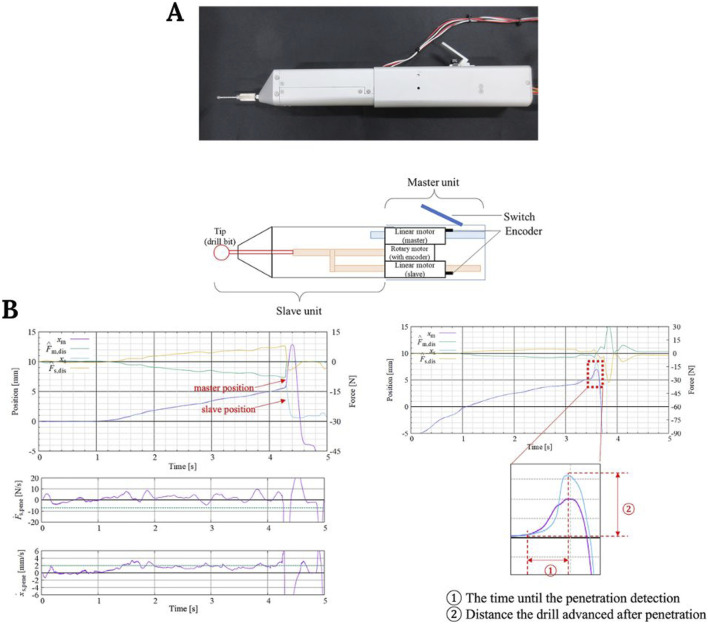
**(A)** Surgical grill and control unit representation. **(B)** Plots showing the time required for penetration detection and the distance traveled during that time. Image modified from Yamanouchi et al. (2023).

On the other hand, in robotic spine surgery, a recently published study reported the development of a promising surgical drill that integrates a haptic interface (Figure 7A). The authors performed an evaluation test on female pigs to detect the penetration of the posterior lamina. They found that in the absence of haptic feedback, the reaction time until surgeons perceived that they had penetrated the lamina with the drill was 0.10–0.22 s, while in the presence of haptic feedback, this reaction time was reduced to 0.01–0.02 s (Figure 7B). Therefore, the integration of these haptic technologies may provide more safety and accuracy in spinal robotic surgeries (Yamanouchi et al., 2023).

### 5.4 Cardiovascular surgery

In cardiovascular surgery, controlling the amount of pressure exerted on the blood vessels is key for a successful intervention. For this reason, cardiovascular interventions constitute another category in which robotic approaches may be beneficial. For instance, some authors developed a master-slave robot for vascular interventions where catheters and guidewire insertions are needed. They aimed to create a steerable catheter with haptic feedback to minimize the risk of vessel damage and the time of radiation exposure (Woo et al., 2019).

### 5.5 Ophthalmological surgery

Intraocular surgery is one of the disciplines of microsurgery and requires precise motor skills for safe outcomes since many small and delicate structures are involved. To master some fine maneuvers, some authors developed a virtual reality simulator in which surgeons were able to train tasks such as the insertion and refill of an eye implant for intravitreal drug delivery. The system was also equipped with haptic resistance during the implant insertion (Heimann et al., 2021).

Moreover, the absence of tremors in robot surgery is a factor extremely beneficial in these interventions. Some authors developed an eye surgery robot that would allow for to safe performance of micromanipulations. The incorporation of haptic feedback using the Phantom Premium devices (Sensible Technologies, 3D Systems) made it more intuitive and ergonomic (Barthel et al., 2015).

## 6 Future directions of haptic feedback systems in robotic surgery

Despite the great advances that have been witnessed in the field of haptic feedback devices, there are still many areas that require further development and research. For instance, some authors support the idea that coupling haptic systems and current surgical robots may lead to diminished surgical performance and heightened physical fatigue during interventions. In this regard, ergonomics is a concern when the surgical team is presented with large devices that might interfere with usual hand movements, either because of their dimensions or their weight (Colan et al., 2024).

Furthermore, in some clinical applications in which the appropriate distinction between the different layers of tissue is needed, such as needle insertions, current challenges rely on the integration of haptic feedback systems for the identification of changes in force measurements with reduced time delay and less risk of tissue damage (Selim et al., 2024).

In addition, further research is still required to recreate tactile sensations more accurately through haptic feedback systems. Multimodal approaches combining kinaesthetic and tactile feedback seem promising, but the determination of the best combination of these modalities is still a challenge to be tackled in the future to achieve a natural palpation feeling (Abiri et al., 2019b; Colan et al., 2024). All these technological advances should be coupled with training programs to teach and improve robotic surgical skills among residents and surgeons.

Recent artificial intelligence (AI) developments can also be applied to haptic feedback systems. The integration of AI models with robotic surgical systems would not only improve efficiency and accuracy by being involved in tasks such as the identification of anatomical structures or path planning through tasks, but also magnify the user’s sense of touch according to the mechanical properties of the tissues (Kella et al., 2025; Sun et al., 2022). In addition to this, some authors are currently working on machine learning models that can also be used to interpolate and infer the magnitude of haptic forces according to measured deformations (Sun and Martius, 2019) and achieve stable haptic rendering in real time for teleoperation applications (Sun et al., 2024).

## 7 Conclusion

Laparoscopic simulators constitute an interesting resource for medical training and preoperative rehearsal. Current bioengineering approaches aim to improve training programs and surgical outcomes, especially with the integration of haptic feedback systems, contributing to more realistic interaction and training. Although novel studies are being carried out to develop haptic feedback integrations for both laparoscopic simulators and surgical robots, further research is still needed to achieve bioinspired artificial palpation that is both affordable and realistic.
